# A nomogram for predicting survival of head and neck mucosal melanoma

**DOI:** 10.1186/s12935-021-01927-7

**Published:** 2021-04-17

**Authors:** Qing-Qing Xu, Qing-Jie Li, Liu Chen, Xin-Yi Su, Jing-Xia Song, Juan Du, Lei Chen, Li-Xia Lu

**Affiliations:** 1 Department of Radiation Oncology, State Key Laboratory of Oncology in South China, Collaborative Innovation Center for Cancer Medicine, Guangdong Key Laboratory of Nasopharyngeal Carcinoma Diagnosis and Therapy, Sun Yat-Sen University Cancer Center, 651 Dongfeng Road East, Guangzhou, 510060 China; 2 Department of Radiation Oncology, Hubei Cancer Hospital, Tongji Medical College, Huazhong University of Science and Technology, Wuhan, 430079 Hubei China

**Keywords:** Mucosal melanoma of the head and neck, Clinical outcomes, Prognostic factors, Nomogram

## Abstract

**Objectives:**

We aimed to understand the clinical characteristics and better predict the prognosis of patients with mucosal melanoma of the head and neck (MMHN) using a nomogram.

**Methods:**

Three hundred patients with nometastatic MMHN were included. Multivariable Cox regression was performed to analyze independent prognostic factors for overall survival (OS), disease-free survival (DFS), distant metastasis-free survival (DMFS), and locoregional relapse-free survival (LRRFS), and these factors were used to develop a nomogram. Concordance indexes (C-indexes), calibration plots, and receiver operating characteristic (ROC) analysis were performed to test the predictive performance of the nomogram in both the primary (n = 300) and validation cohorts (n = 182).

**Results:**

The primary tumor site, T stage and N stage were independent risk factors for survival and were included in the nomogram to predict the 3- and 5-year OS, DFS, DMFS, and LRRFS in the primary cohort. The C-indexes (both > 0.700), well-fit calibration plots, and area under the ROC curve (both > 0.700) indicated the high diagnostic accuracy of the nomogram, in both the primary and validation cohorts. The patients were divided into three groups (high-risk, intermediate-risk, and low-risk groups) according to their nomogram scores. The survival curves of OS, DFS, DMFS, and LRRFS were well separated by the risk groups in both cohorts (all P < 0.001).

**Conclusions:**

The nomogram can stratify MMHN patients into clinically meaningful taxonomies to provide individualized treatment.

## Introduction

Mucosal melanoma of the head and neck (MMHN) is a highly malignant tumor accounting for more than 50% of all mucosal melanomas [1] and is predominantly found in the nasal cavity, paranasal sinuses and oral cavity [2]. Compared with cutaneous and acral melanomas, mucosal melanoma has aggressive clinical characteristics and a worse prognosis [3, 4]. Despite the application of various clinical treatments, including surgery, radiotherapy, chemotherapy and immunotherapy, the local control and long-term prognosis of MMHN remain dismal and have shown no improvement trends in recent years [5, 6]. 

Many studies have focused on exploring the prognostic value of common clinical diagnostic methods and using these characteristics of diagnostic results to construct a relatively excellent prognostic prediction model [7, 8]. For example, logistic regression models, based on age, gender, number of PET-CT examinations, and occupational exposure, were performed to analyze cancer mortality and identify the major risk factors [7]. Second-generation sequencing data combined with the clinical information of the patients with chronic lymphocytic leukemia reflecting the tumor heterogeneity of patients were helpful to predict the prognosis [8]. A nomogram serves as a new reliable tool for predicting the prognosis by including variables to analyze their effects on survival [9]. Moreover, nomograms have been widely used in clinical work, such as to predict the benefit of neoadjuvant chemoradiotherapy for esophageal cancer [10], radiotherapy or adjuvant chemoradiotherapy for resected gallbladder cancer [11]. However, because of its rarity, no effective prognostic tool is available for MMHN, making individualized treatment difficult to achieve. Therefore, more accurate prognostic tools are needed to determine the malignant degree of MMHN and optimize treatment.

In this study, we combined the TNM staging system and clinical features to develop and validate a nomogram to accurately predict the survival outcomes of MMHN patients and facilitate clinical decision making.

## Materials and methods

### Patients

**Fig. 1:**
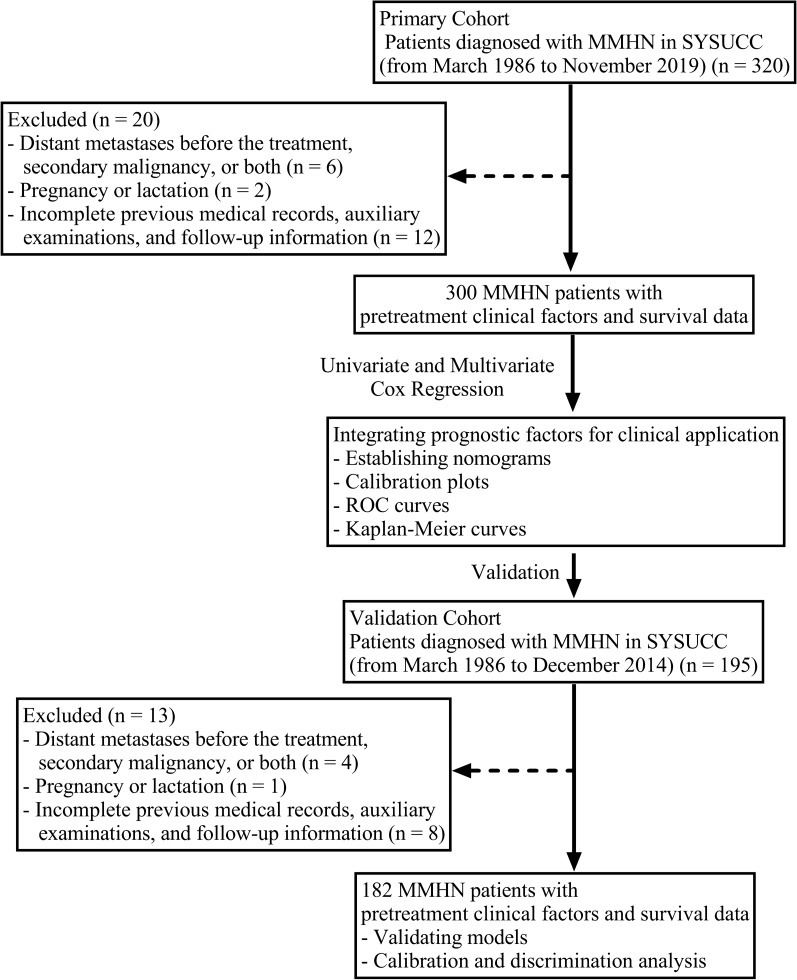
Flow chart of the study design. MMHN, mucosal melanoma of the head and neck; SYSUCC: Sun Yat-Sen University Cancer Center; ROC: receiver operating characteristic

The study workflow is displayed in Fig. 1. From March 1986 to November 2019, three hundred patients diagnosed with MMHN at our hospital were included in the primary cohort. MMHN patients from March 1986 to December 2014 were chosen as the validation cohort. The inclusion criteria for all the patients were previously untreated, nonmetastatic, and newly histologically confirmed stage III-IVB MMHN. All the patients were restaged according to the 8th edition American Joint Committee on Cancer (AJCC) staging system for MMHN [12]. The TNM classification was based on surgical documents, pathological features and imaging findings. The exclusion criteria were as follows: (1) distant metastases before treatment, secondary malignancy, or both; (2) pregnancy or lactation; and (3) incomplete previous medical records, auxiliary examinations, and follow-up information. The Ethics Committee at Sun Yat-sen University Cancer Center in China approved our study protocol.

### Follow-up and endpoints

Our main endpoint was overall survival (OS), and the secondary endpoints were disease-free survival (DFS), distant metastasis-free survival (DMFS), and locoregional relapse-free survival (LRRFS). OS was defined as the time from the diagnosis of melanoma to the date of death or the last known follow-up, whichever occurred first. DMFS was defined as the time to distant metastasis, death, or patient censoring, whichever occurred first. DFS was defined as the time to failure, death from any cause, or patient censoring, whichever occurred first. LRRFS was defined as the time to local/regional relapse, death, or patient censoring, whichever occurred first. The median follow-up time was 32 months (range: 1–262 months). After treatment, the patients were evaluated once every 3 months during the first 3 years and every 6 months thereafter. 

### Statistical analysis

The patients were classified into two groups based on age (< 60 years vs. ≥ 60 years). Variables satisfying *P* < 0.1 in univariate Cox regression analysis were included in multivariable analysis. *P* < 0.05 in multivariable Cox regression analysis was used to select independent prognostic variables of survival. All the independent prognostic factors were used to create a predictive nomogram (by the rms package in R). The concordance index (C-index) values with 95% confidence intervals (CIs) were evaluated to assess the accuracy of the nomogram in the primary and validation cohorts. The calibration plots for OS, DMFS, and DFS at 3 and 5 years were generated by comparing the predicted OS, DMFS, and DFS with the actual OS, DMFS, and DFS. Additionally, the predictive precision and discrimination of the nomogram were further analyzed using C-index, and area under the curve (AUC) of the receiver operating characteristic (ROC) curve. Survival curves were generated using the Kaplan–Meier method. Differences in survival between risk groups were analyzed by the log-rank test. Statistical analysis was performed using R software (R version 3.6.1) (http://www.r-project.org) and IBM SPSS software version 25.0.

## Results

### Patient characteristics and follow-up

From March 1986 to November 2019, 300 patients (primary cohort) and 182 patients (validation cohort) with MMHN were enrolled in this study. The median age was 57 (range, 19–87) years, with a male-to-female ratio of 1.54:1 for the primary cohort. The median age was 58 (range, 25–80) years, with a male-to-female ratio of 1.54:1 for the validation cohort. The baseline patient characteristics of the primary cohort and validation cohort are listed in Table 1.

**Table 1:** Comparison of the different characteristics between patients in the primary and validation cohorts

Characteristic	Number of patients (%)		*P*-value
	Primary patients (n = 300)	Validation cohort (n = 182**)**	
Gender			0.944
Male	182 (60.7)	111 (61.0)	
Female	118 (39.3)	77 (39.0)	
Age (years old)			0.373
< 60	174 (58.0)	98 (53.8)	
≥ 60	126 (42.0)	84 (46.2)	
Smoking			0.064
No	220 (73.3)	119 (65.4)	
Yes	80 (26.7)	63 (34.6)	
Primary site			0.753
Others	44 (14.7)	23 (12.6)	
Nasal cavity	159 (53.0)	100 (54.9)	
Paranasal sinus	24 (8.0)	11 (6.0)	
Oral cavity	73 (24.3)	48 (26.4)	
8th T stage			0.966
T3	121 (40.3)	73 (40.1)	
T4a	129 (43.0)	77 (42.3)	
T4b	50 (16.7)	32 (17.5)	
8th N stage			0.604
N0	224 (74.7)	132 (72.5)	
N1	76 (25.3)	50 (27.5)	
Surgery			0.933
No	21 (7.0)	13 (7.1)	
Yes	170 (56.7)	100 (54.9)	
Reoperation	109 (36.3)	69 (37.9)	
Radiotherapy			** < 0.001**
No	179 (59.7)	129 (70.9)	
2D	22 (7.3)	22 (12.1)	
IMRT	99 (33.0)	31 (17.0)	
Chemotherapy			0.377
No	131 (43.7)	87 (47.8)	
Yes	169 (56.3)	95 (52.2)	
Immunotherapy			0.659
No	217 (72.3)	135 (74.2)	
Yes	83 (27.7)	47 (25.8)	

All continuous variables were changed to categorical variables. Pearson χ^2^ test was used to compute the *P*-value

The median follow-up time was 32.0 months (range, 1–262 months). In total, 172 patients had disease failure; 75 (7.7%), 52 (3.8%), and 109 (14.6%) patients developed local recurrence, regional recurrence, and distant metastasis, respectively; 188 (14.4%) patients died.

### Univariate and multivariate Cox regression analyses

**Fig. 2:**
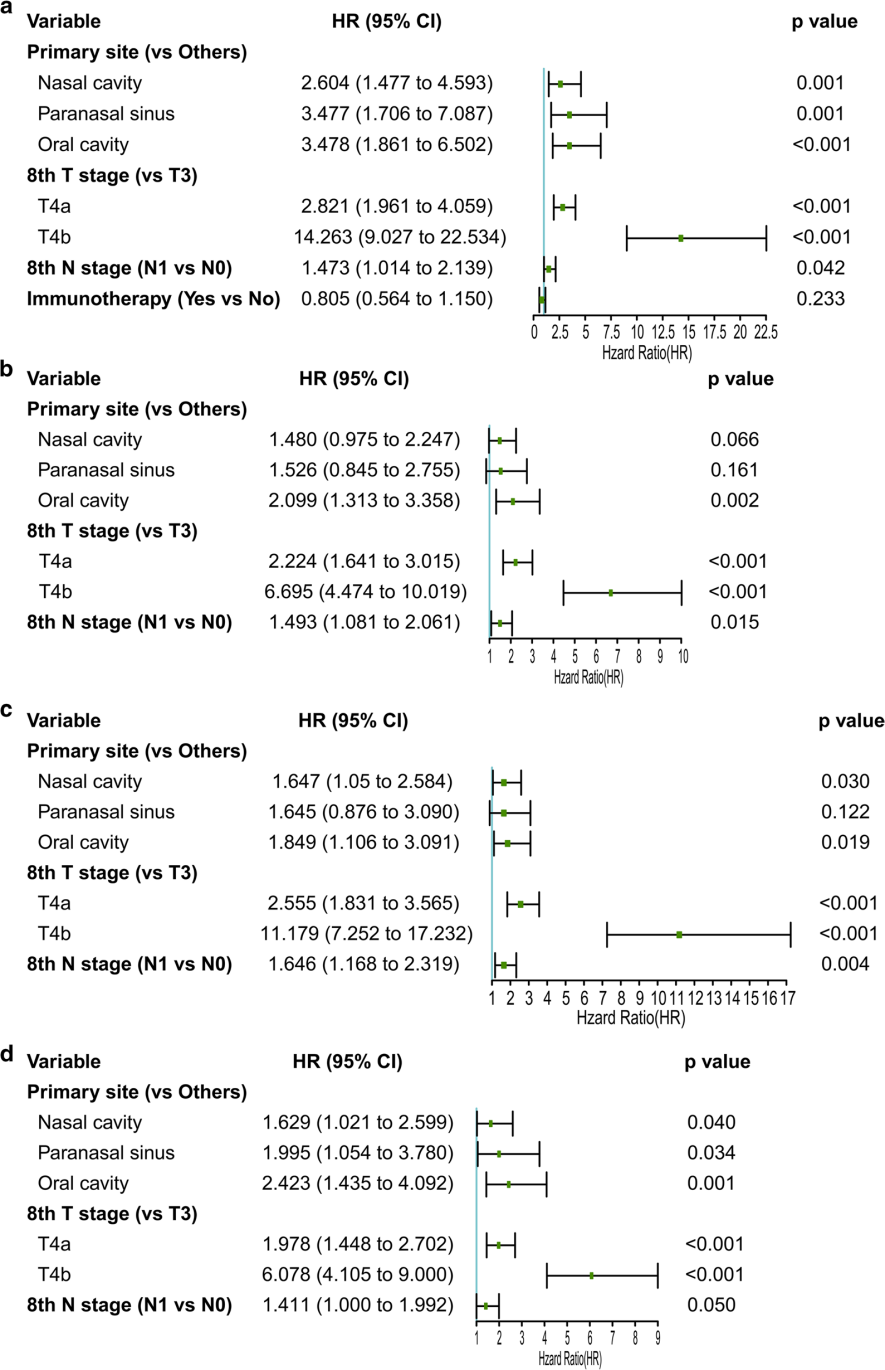
Multivariate Cox regression analysis of OS (**a)**, DFS (**b)**, DMFS (**c)**, and LRRFS (**d)** in the primary cohort

The significant variables related to OS were primary tumor location, T stage, N stage, and immunotherapy in univariate analysis. The significant variables related to DFS, DMFS, and LRRFS were primary tumor location, T stage, and N stage in univariate analysis. We incorporated the above factors into multivariate Cox regression analysis. Eventually, T stage, N stage, and the primary tumor site were independent prognostic factors. The results of univariate and multivariate Cox analyses are summarized in Tables 2 and 3 and Fig. 2.

**Table 2:** Univariate Cox regression analysis of OS and DFS in the primary cohort

Variable	OS		DFS	
	HR (95% CI)	*P*	HR (95% CI)	*P*
Gender				
Male	Reference		Reference	
Female	0.964 (0.717–1.297)	0.811	1.051 (0.766–1.442)	0.759
Age (years old)				
< 60	Reference		Reference	
≥ 60	1.246 (0.934–1.660)	0.134	1.203 (0.881–1.644)	0.245
Smoking				
No	Reference		Reference	
Yes	1.020 (0.747–1.395)	0.899	0.787 (0.591–1.049)	0.102
Primary site				
Others	Reference		Reference	
Nasal cavity	2.127 (1.256–3.603)	0.005^**^	1.510 (0.996–2.288)	**0.052**
Paranasal sinus	6.113 (3.101–12.052)	< 0.001^***^	3.066 (1.733–5.425)	< 0.001^***^
Oral cavity	2.586 (1.465–4.564)	0.001^**^	2.217 (1.413–3.479)	0.001^**^
8th T stage				
T3	Reference		Reference	
T4a	2.801 (1.968–3.989)	< 0.001^***^	2.150(1.596–2.896)	< 0.001^***^
T4b	11.025(7.301–16.647)	< 0.001^***^	6.179 (4.227–9.034)	< 0.001^***^
8th N stage				
N0	Reference		Reference	
N1	1.542 (1.113–2.237)	0.009^**^	1.637 (1.223–2.191)	0.001^**^
Surgery				
No	Reference		Reference	
Yes	0.707 (0.409–1.220)	0.212	0.612 (0.386–0.969)	0.036^*^
Radiotherapy				
No	Reference		Reference	
Yes	1.178 (0.879–1.580)	0.273	1.070 (0.828–1.383)	0.603
Chemotherapy				
No	Reference		Reference	
Yes	0.863 (0.647–1.151)	0.317	1.022 (0.792–1.318)	0.869
Immunologic/targeted therapy				
No	Reference		Reference	
Yes	0.694 (0.495–0.974)	0.035^*^	0.873 (0.657–1.159)	0.348

Cox proportional hazard model was used to conduct Cox regression analysis. * p value < 0.05; ** p value < 0.01; ** p value < 0.001

**Table 3:** Univariate Cox regression analysis of DMFS and LRRFS in the primary cohort

Variable	DMFS		LRRFS	
	HR (95% CI)	*P*	HR (95% CI)	*P*
Gender				
Male	Reference		Reference	
Female	0.964 (0.717–1.297)	0.811	1.036 (0.793–1.355)	0.794
Age (years old)				
< 60	Reference		Reference	
≥ 60	1.246 (0.641–1.164)	0.134	1.070 (0.822–1.391)	0.616
Smoking				
No	Reference		Reference	
Yes	0.864 (0.625–1.236)	0.336	0.819 (0.609–1.100)	0.185
Primary site				
Others	Reference		Reference	
Nasal cavity	1.695 (1.084–2.649)	0.021^*^	1.795 (1.128–2.858)	0.014^*^
Paranasal sinus	3.736 (2.032–6.867)	< 0.001^***^	3.710 (1.995–6.897)	< 0.001^***^
Oral cavity	1.9844 (1.217–3.235)	0.006^**^	2.631 (1.601–4.323)	< 0.001^***^
8th T stage				
T3	Reference		Reference	
T4a	2.544(1.838–3.520)	< 0.001^***^	1.945 (1.434–2.639)	< 0.001^***^
T4b	10.314(6.861–15.502)	< 0.001^***^	5.816 (4.002–8.452)	< 0.001^***^
8th N stage				
N0	Reference		Reference	
N1	1.597 (1.180–2.161)	0.002^**^	1.665 (1.231–2.253)	0.001^***^
Surgery				
No	Reference		Reference	
Yes	0.758 (0.455–1.262)	0.286	0.664 (0.409–1.078)	0.098
Radiotherapy				
No	Reference		Reference	
Yes	1.359 (1.037–1.224)	0.026^*^	1.007 (0.771–1.316)	0.957
Chemotherapy				
No	Reference		Reference	
Yes	1.022 (0.792–1.318)	0.869	0.934 (0.718–1.215)	0.611
Immunologic/targeted therapy				
No	Reference		Reference	
Yes	0.858 (0.634–1.161)	0.320	0.801 (0.594–1.079)	0.144

Cox proportional hazard model was used to conduct Cox regression analysis. * p value < 0.05; ** p value < 0.01; ** p value < 0.001

### Establishing and validating a nomogram

**Fig. 3:**
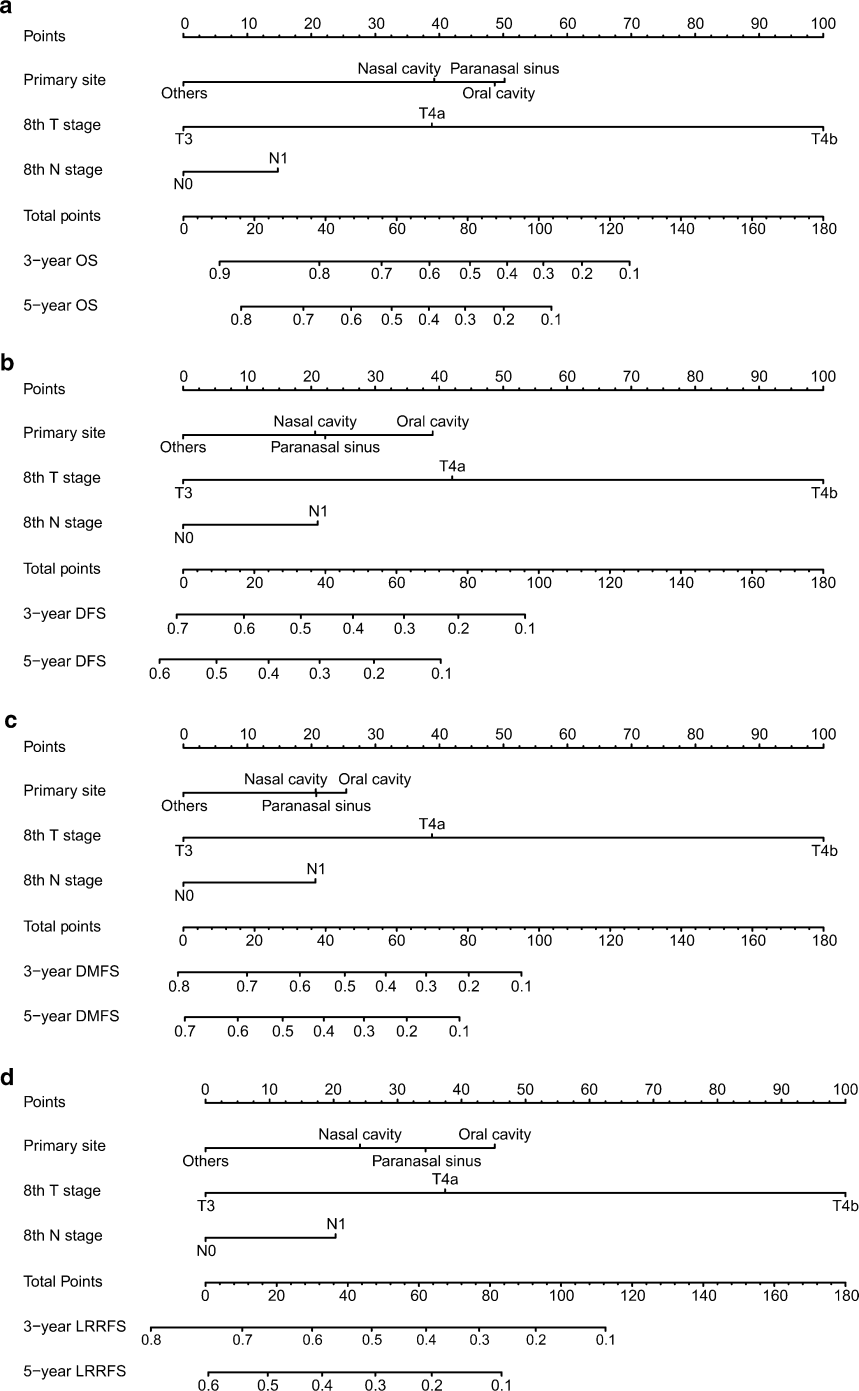
Developing a nomogram. The nomogram was based on the NPC patients’ data of the primary site, 8th T stage, and 8th N stage for 3- and 5-year OS (**a)**, DFS (**b)**, DMFS (**c)**, and LRRFS (**d)** in the primary cohort

All the factors, including the primary tumor site, T stage, and N stage, were included in the nomogram. By summarizing the score of each variable and positioning the total scores on the score scale, a nomogram was established to predict the 3- and 5-year OS, DFS, DMFS, and LRRFS in the primary cohort (Fig. 3a–d).

The prognostic usefulness of the radiomic features was assessed using the concordance indexes (C-indexes). A C-index of 1 indicates perfect concordance, and a C-index of 0.5 corresponds to random chance. The C-indexes of the nomogram for predicting OS, DFS, DMFS, and LRRFS were almost greater than 0.7 in all the cohorts, indicating that the performance of the model was satisfactory.

**Fig. 4:**
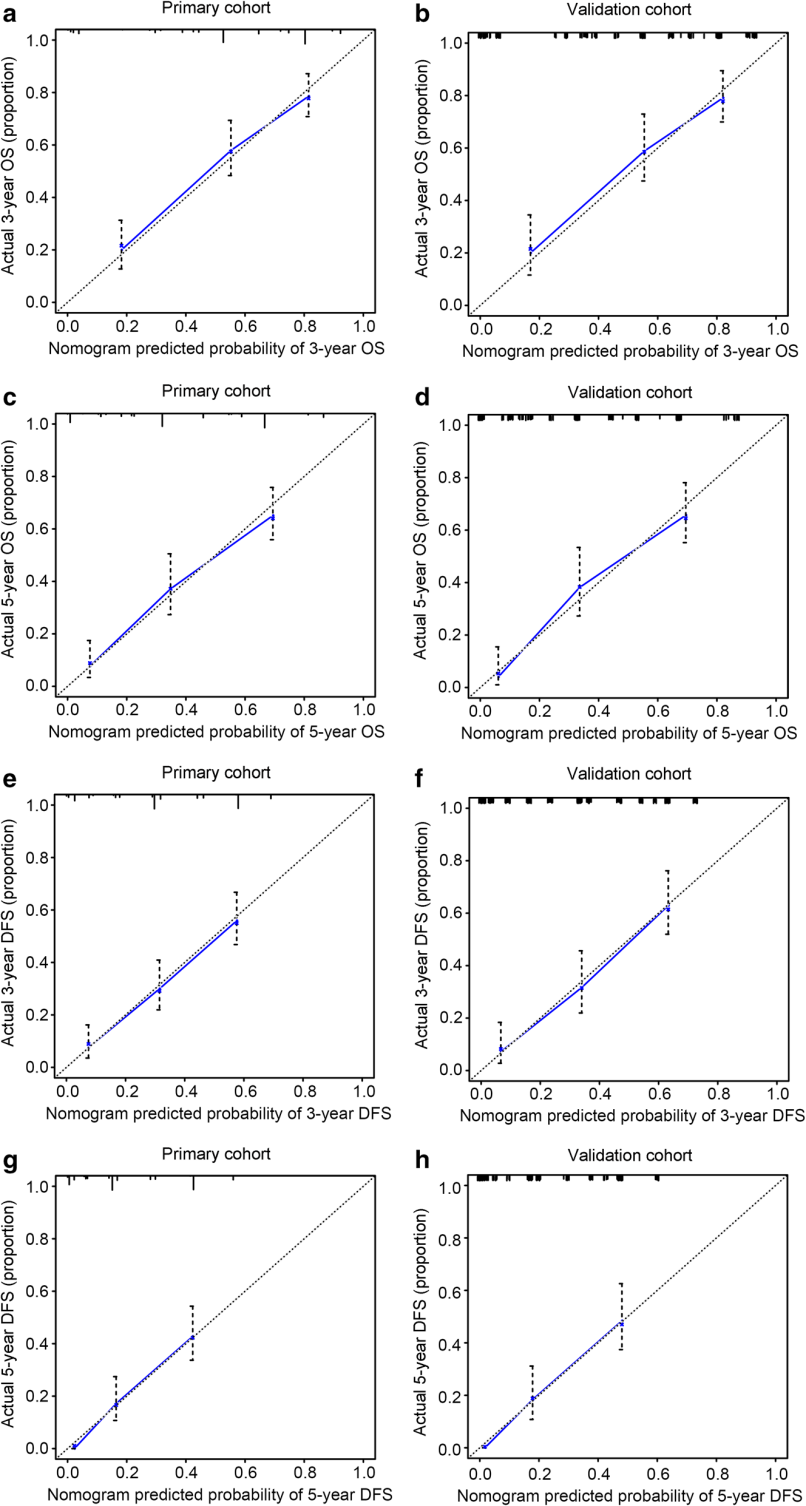
Calibration plots of the nomogram to predict the 3- and 5-year OS and DFS in the primary (**a**, **c**, **e**, **g)** and validation cohorts (**b,** **d**, **f**, **h**). Nomogram-estimated 3- or 5-year OS (**a–d**) and DFS (**e–h**) were plotted on the x-axis; the observed OS and DFS were plotted on the y-axis. Dashed lines along the 45-degree line represented that the predicted probabilities are equal to the actual probabilities

**Fig. 5:**
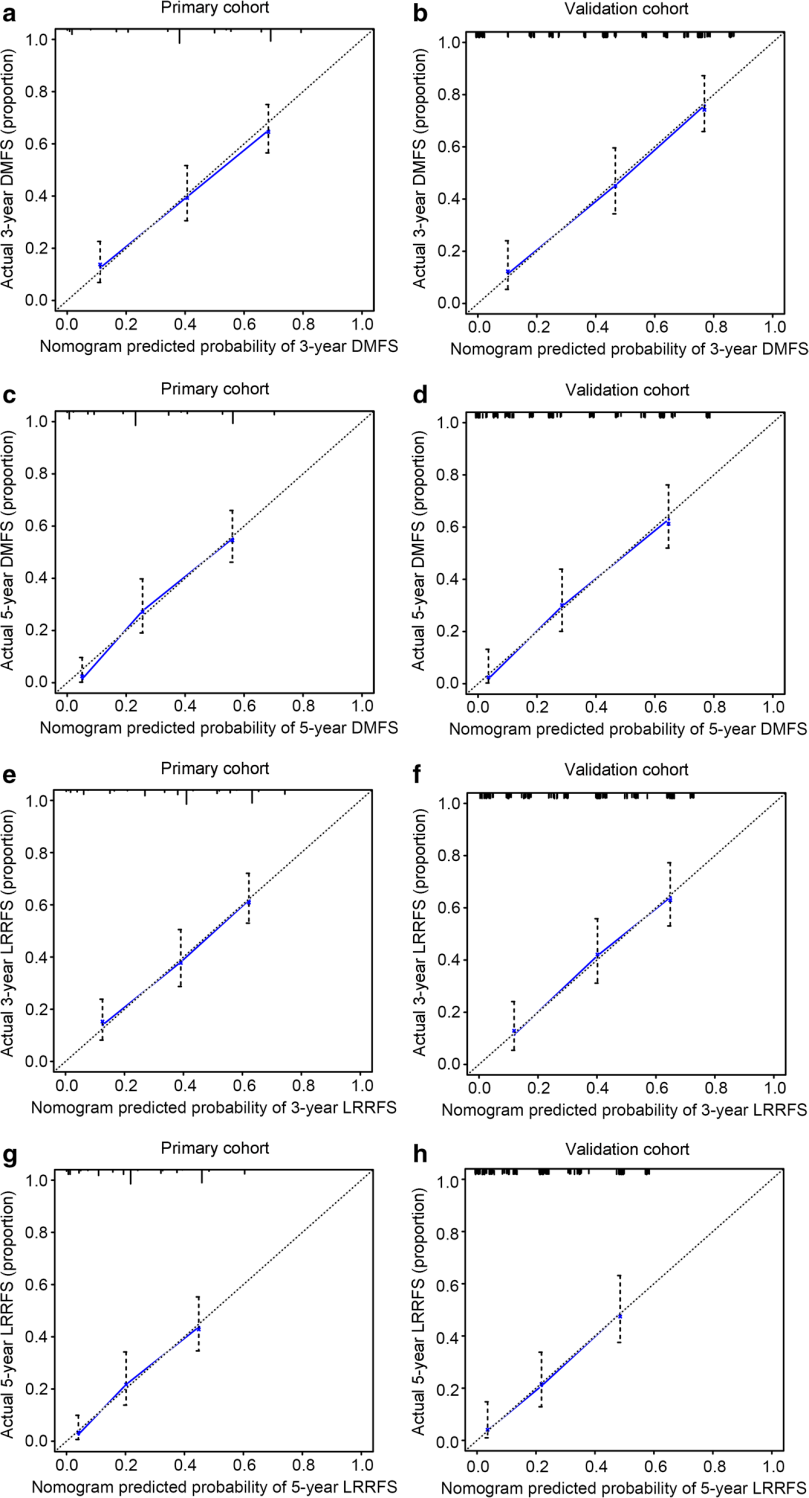
Calibration plots for the 3- and 5-year DMFS and LRRFS in the primary (**a**, **c**, **e**, **g**) and validation cohorts (**b**, **d**, **f**, **h**). The nomogram-estimated 3- or 5-year DMFS (**a–d**) and LRRFS (**e–h**) were plotted on the x-axis; the observed DMFS and LRRFS were plotted on the y-axis. The dashed lines along the 45-degree line representing the predicted probabilities equal the actual probabilities

**Fig. 6:**
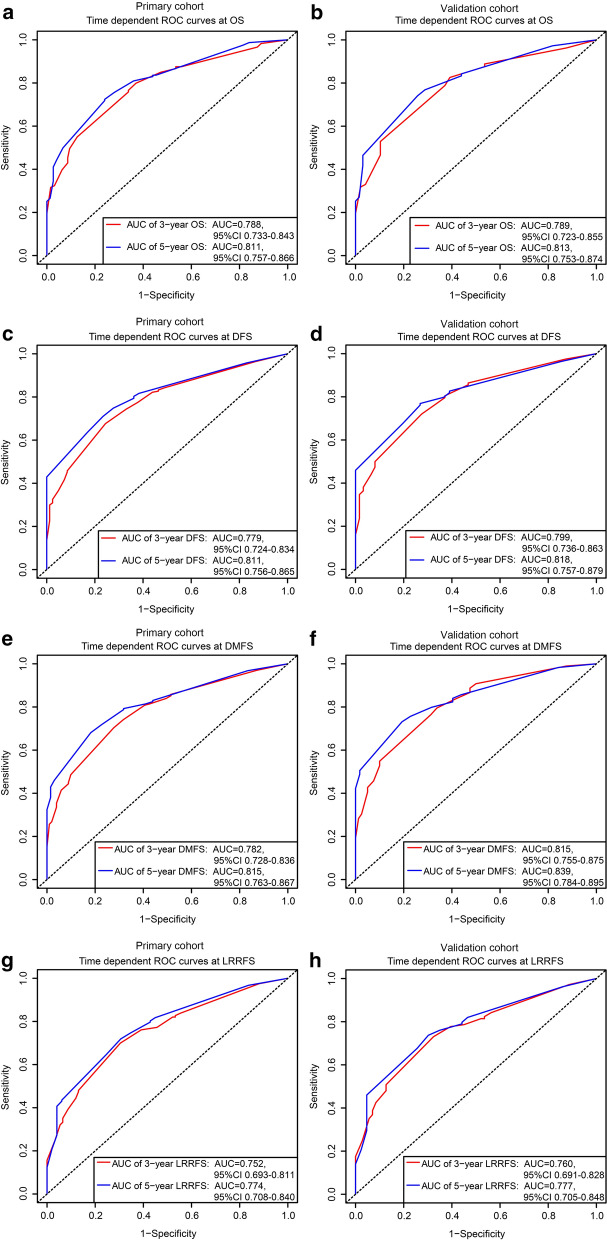
ROC curves by the nomogram to predict the 3- and 5-year OS (**a**, **b**), DFS (**c**, **d**), DMFS (**e**, **f**), and LRRFS (**g**, **h**)

In the calibration plot, the X-axis represents the nomogram prediction of OS, DFS, DMFS, and LRRFS, and the Y-axis represents the observed values of OS, DFS, DMFS, and LRRFS calculated by the Kaplan–Meier method. The solid line represents the ideal reference line, indicating the consistency between the predicted survival rate and observed survival rate. The calibration plots for the OS (Fig. 4a–d) and DFS (Fig. 4e–h) probabilities at 3 and 5 years all approached 45 degrees. These results indicated that the predicted survival rates of 3 and 5 years agreed strongly with the actual survival rates. Furthermore, the calibration curves for DMFS (Fig. 5a–d) and LRRFS (Fig. 5e–h) probabilities at 3 and 5 years showed a significant association between the predictions and observations in all cohorts. ROC curve analysis was also used to assess the predictive capacity of the nomogram. The area under the ROC curve (AUC) represents the accuracy of the diagnostic methods; ROC-AUC = 0.50 indicates no discrimination, and ROC-AUC = 1.0 indicates perfect discrimination. In this nomogram model, the AUCs for the 3- and 5-year OS reached 0.788 and 0.811 in the primary cohort and 0.789 and 0.813 in the validation cohort, respectively (Fig. 6a, b). The ROC curves for the 3- and 5-year DFS, DMFS, and LRRFS also showed an excellent predictive value (AUC > 0.70 in all cohorts) (Fig. 6c–h). These results revealed that the nomogram for predicting survival could effectively screen out high-risk MMHN patients with a relatively worse survival.

### Nomogram for risk stratification

**Fig. 7:**
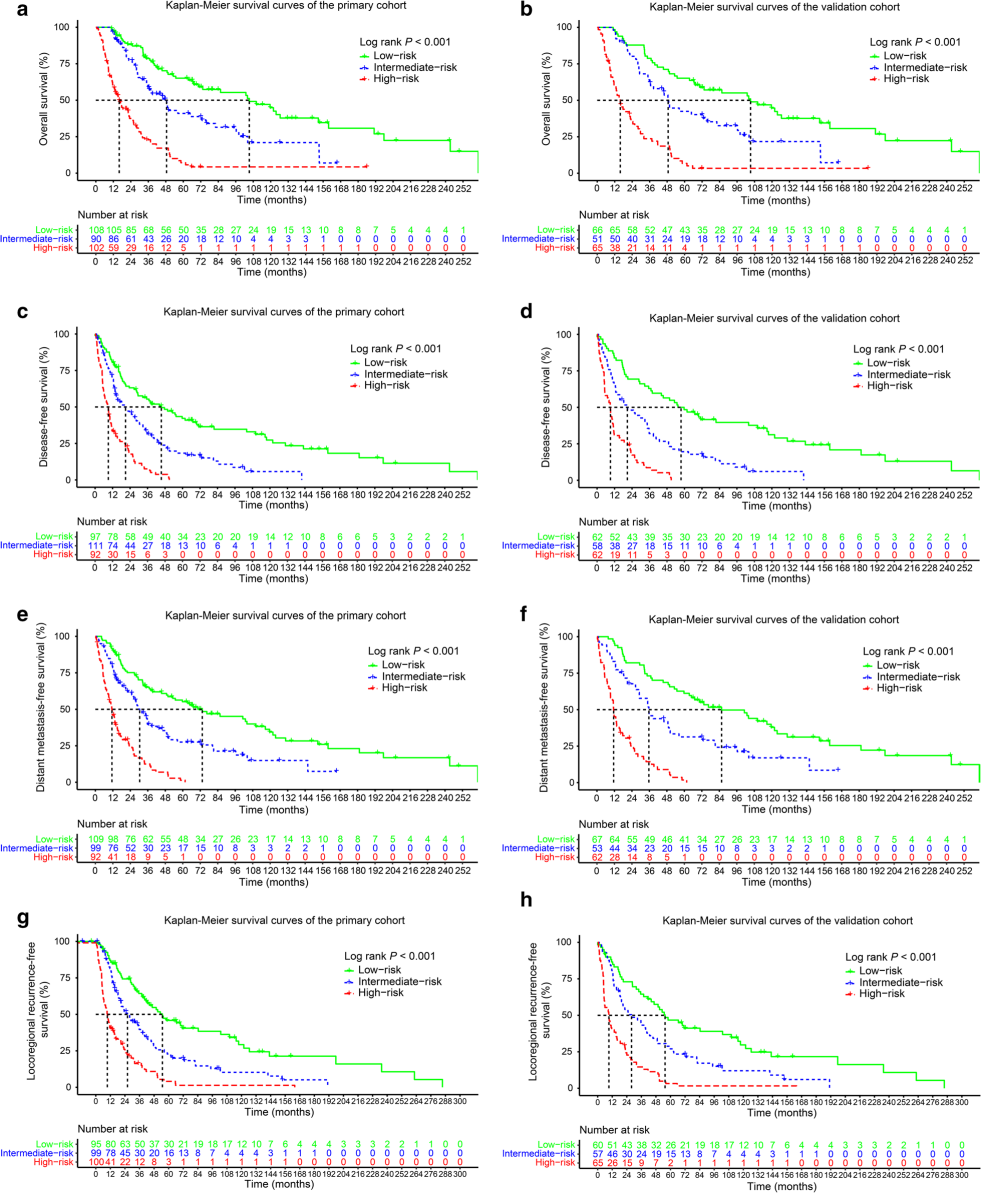
Kaplan–Meier curves for OS (**a**, **b**), DFS (**c**, **d**), DMFS (**e**, **f**), and LRRFS (**g**, **h**) of patients in the low-, intermediate- and high-risk groups. The risk groups were stratified according to the 33% and 66% of total risk scores in the primary (**a**, **c**, **e**, **g**) and validation cohorts (**b**, **d**, **f**, **h**)

Consequently, stratification was established according to the nomogram for OS, DFS, DMFS, and LRRFS. Based on the total scores calculated by the nomogram, we set a threshold for the total score (33% and 66%). The primary and validation cohorts were divided into low-risk [total score: < 50 (OS); < 42 (DFS); < 40 (DMFS); < 45 (LRRFS)], intermediate-risk [total score: 50–85 (OS); 42–64 (DFS); 40–60 (DMFS); 45–69 (LRRFS)] and high-risk [total score: > 85 (OS); > 64 (DFS); > 60 (DMFS); > 69 (LRRFS)] groups. According to the Kaplan–Meier plots, the MMHN patients in the high-risk group exhibited a worse OS (Fig. 7a, b), DFS (Fig. 7c, d), DMFS (Fig. 7e, F), and LRRFS (Fig. 7g, h) than those in the low-risk group (all P-values < 0.001). Taken together, the results suggest that our nomogram is an effective predictor of the prognosis of MMHN. This tool may help to provide significant clinical implications for the personalized therapy of MMHN patients.

**Fig. 8:**
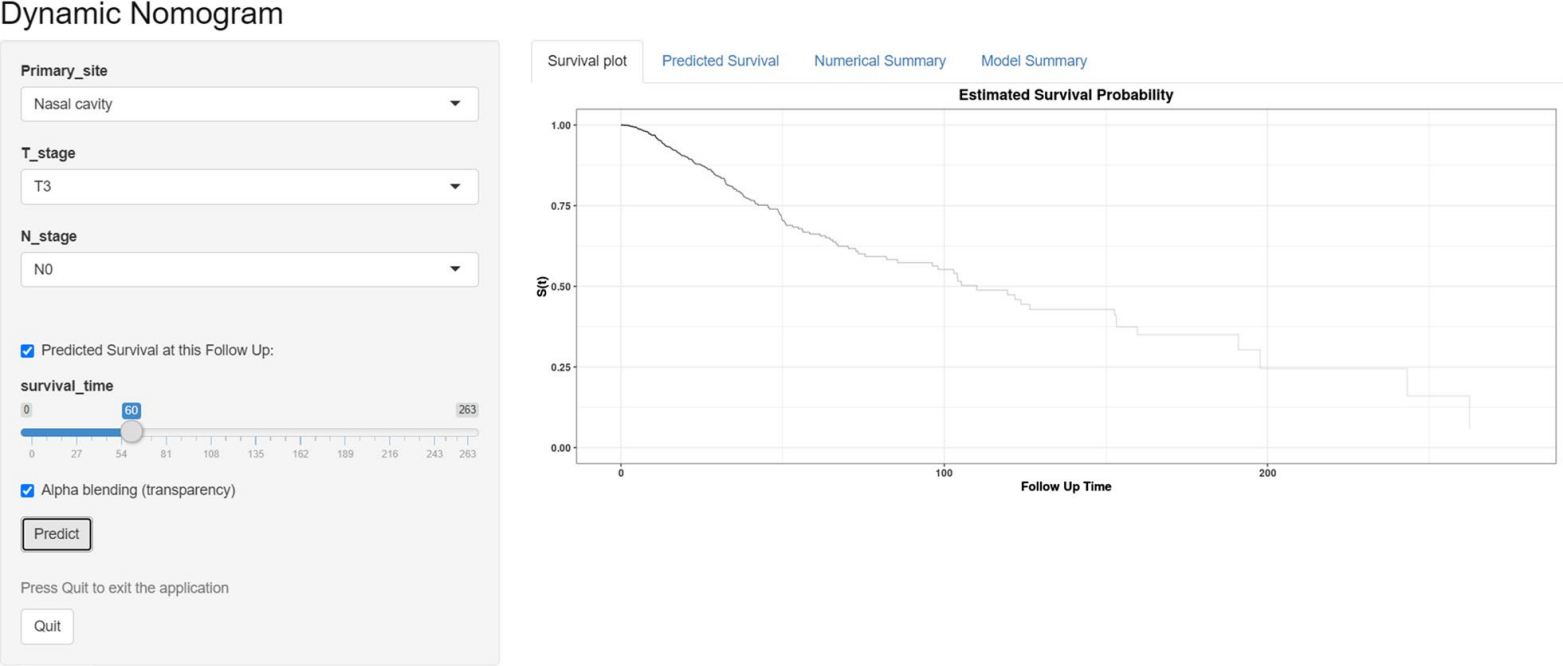
Establishing web-based calculators according to the nomograms

Establishing web-based calculators according to the nomograms. We have created web servers for our nomograms (Fig. 8) that can be easily accessed at https://liling.shinyapps.io/MMHN_OS/, https://liling.shinyapps.io/MMHN_DFS/, https://liling.shinyapps.io/MMHN_DMFS/, and https://liling.shinyapps.io/MMHN_LRRFS/. The OS, DFS, DMFS, and LRRFS in NPC patients can be predicted conveniently by selecting corresponding clinical features and reading the generated figures and tables.

## Discussion

MMHN is an aggressive malignancy whose comprehensive treatment modalities are complex and clinical outcomes remain unsatisfactory [13]. The AJCC 8th edition staging system cannot predict prognosis [14]. MMHN patients in the same stage can have obviously different survival rates [15]. Previous studies had reported that integrating clinical factors to construct predictive models is beneficial to clinical decision making [16, 17]. For example, CT image parameters in the head and neck region were added to build a model to determine the CT absorbed dose distribution of routine scan parameters in adults [16]. Accurate assessment of the risk of a secondary cancer due to radiotherapy was performed according to dose distributions in the exposure field detected by Markus ionization chamber detectors [17]. In the present study, we integrated other clinical factors into the TNM staging system, adding prognostic value to the TNM system to help clinicians guide more promising treatment decisions.

Our findings reveal that the primary tumor site is an independent prognostic factor. Consistent with the findings of several previous studies, our results showed worse outcomes when the primary tumor site was in the paranasal cavity than in the oral or nasal cavities [18, 19]. Several plausible reasons may explain the poorer survival of patients with a primary tumor site in the paranasal sinuses. Because of the complex anatomical location of the paranasal sinus, paranasal sinus melanoma is found at later stages and has a wide range of invasion; thus, the prognosis of these patients may be poor. T stage and N stage were also independent prognostic factors, similar to the findings of other articles [20, 21]. Several studies have revealed a nomogram of the lymph node status in patients with melanoma or a nomogram of melanoma site [22, 23]. However, to our best knowledge, no nomogram was constructed to predict survival in patients with MMHN. Our study also combined the primary site with T stage and N stage to construct a nomogram. The predictive ability of our nomogram is relatively high. For example, the C-indexes and AUCs of the nomogram for predicting OS, DFS, DMFS, and LRRFS were almost greater than 0.7 in all cohorts.

The study has the following advantages. First, the survival rate of a patient can be calculated simply and visually. For example, a patient with T3 (0 points) N0 (0 points) paranasal sinus MM (50 points) who visits the clinic would have a total of 50 points, yielding an estimated 3-year OS rate of 28.57%. Second, the nomogram can further divide patients into high-, intermediate- and low-risk groups, and the survival rate of each group can effectively distinguish the prognosis. Doctors can prescribe individualized treatment to patients according to their corresponding groups. The intensity of treatment can be appropriately strengthened for patients in the high-risk group. For example, immunologic/targeted therapy can improve the survival rate of MMHN [24]. Finally, in addition to the traditional nomogram, we also created a dynamic nomogram that can predict the prognosis of patients through simple operation on a web page (Fig. 8).

Our study has several limitations. First, this study was retrospective with inevitable selection bias. However, this retrospective study was worth performing because it crucial to lay the foundation for further prospective studies.

Second, this study involved patients from the same hospital; thus, the findings may lack applicability for patients from other regions and institutions. Finally, the sample size was not large enough; thus, no significant differences were found in some prognostic factors, particularly the treatment modalities. However, our study included 300 Chinese MMHN patients, which currently constitutes the largest sample from a single center to our knowledge.

In conclusion, the study developed and validated a nomogram based on the primary tumor site, T stage, and N stage to predict OS, DFS, DMFS, and LRRFS in MMHN patients, so as to help clinicians identify high-risk patients and make individualized treatment.

## Data Availability

The databases analyzed during the current study are available.

## Abbreviations

AJCC/UICC: American Joint Committee on Cancer/Union for International Cancer control
AUC: Area under the curve
C-index: Concordance index
CI: Confidence interval
DFS: Disease-free survival
DMFS: Distant metastasis-free survival
HR: Hazard ratio
LRRFS: Locoregional relapse-free survival
MMHN: Mucosal melanoma of the head and neck
OS: Overall survival
ROC: Receiver operating characteristic
IMRT: Intensity-modulated radiotherapy
